# Functionalized Mesoporous Silicon Nanomaterials in Inorganic Soil Pollution Research: Opportunities for Soil Protection and Advanced Chemical Imaging

**DOI:** 10.1007/s40726-020-00152-6

**Published:** 2020-07-01

**Authors:** Jia-Wei Yang, Wen Fang, Paul N. Williams, John W. McGrath, Carlos Eduardo Eismann, Amauri Antonio Menegário, Lucas Pellegrini Elias, Jun Luo, Yingjian Xu

**Affiliations:** 1 Institute for Global Food Security, School of Biological Sciences, Queen’s University Belfast, Belfast, Northern Ireland BT9 5DL UK; 2 State Key Laboratory of Pollution Control and Resource Reuse, School of the Environment, Nanjing University, Nanjing, 210023 Jiangsu China; 3 Environmental Studies Center (CEA), São Paulo State University (UNESP), Avenida 24-A, 1515, Rio Claro, SP 13506-900 Brazil; 4 Department of Chemistry, University of Warwick, Coventry, CV4 7AL UK; 5 GoldenKeys High-Tech Materials Co., Ltd., Building B, Innovation & Entrepreneurship Park, Guian New Area, Guian, 550025 Guizhou China

**Keywords:** Functional mesoporous silicon nanomaterials (FMSN), Diffusive gradient in thin films (DGT), X-ray fluorescence spectrometry (XRF), Heavy metals, Soil pollution

## Abstract

“Innovative actions towards a pollution free-planet” is a goal of the United Nations Environment Assembly (UNEA). Aided by both the Food and Agricultural Organisation (FAO) and its Global Soil Partnership under the 3rd UNEA resolution, a consensus from > 170 countries have agreed a need for accelerated action and collaboration to combat soil pollution. This initiative has been tasked to find new and improved solutions to prevent and reduce soil pollution, and it is in this context that this review provides an updated perspective on an emerging technology platform that has already provided demonstrable utility for measurement, mapping, and monitoring of toxic trace elements (TTEs) in soils, in addition to the entrapment, removal, and remediation of pollutant sources. In this article, the development and characteristics of functionalized mesoporous silica nanomaterials (FMSN) will be discussed and compared with other common metal scavenging materials. The chemistries of the common functionalizations will be reviewed, in addition to providing an outlook on some of the future directions/applications of FMSN. The use of FMSN in soil will be considered with some specific case studies focusing on Hg and As. Finally, the advantages and developments of FMSN in the widely used diffusive gradients-in-thin films (DGT) technique will be discussed, in particular, its advantages as a DGT substrate for integration with oxygen planar optodes in multilayer systems that provide 2D mapping of metal pollutant fluxes at submillimeter resolution, which can be used to measure detailed sediment-water fluxes as well as soil-root interactions, to predict plant uptake and bioavailability.

**Electronic supplementary material:**

The online version of this article (10.1007/s40726-020-00152-6) contains supplementary material, which is available to authorized users.

## Introduction to FMSN

### The Development of FMSN and Its Principles of Adsorption

**Fig. 1:**
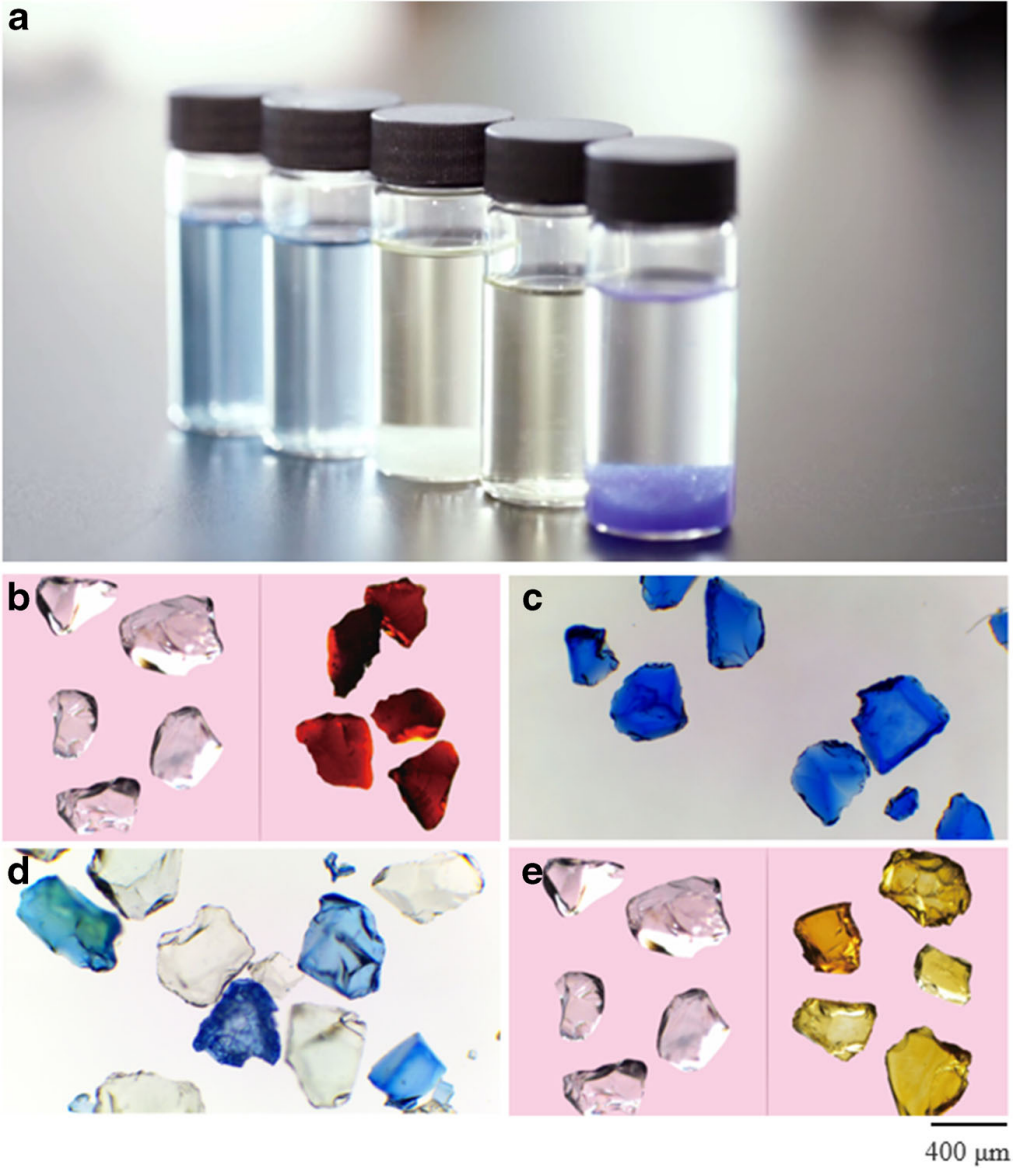
Copper removal by iMoLboX FMSN and its high absorbing capacity. **a** Partitioning of Cu from 15 ml 98% anhydrous ethanol solution (pH 4–6) containing 30 mg L^−1^ CuSO_4_ into 0.025 g FMSN, removal efficiency reached 92.97%. The color change of the solution is caused by bromophenol blue (0.1–0.3 ml). The color of FMSN changed from colorless to blue after the adsorption was completed. **b** Si crystal image before and after palladium recovery from a solution (pH 2–5) of 250 mg L^−1^ PbCl_2_. **c** and **d** Si dosed with 30 mg L^−1^ CuSO_4_ solution (pH 4–6). **e** Si crystal image before and after iron recovery from a solution (pH 3–5) of 150 mg L^−1^ FeCl_3_/FeCl_2_. Si crystal particle size is 180–600 microns. The images of FMSN particle were obtained through compound optical microscope (LEICA DM500), provided by GoldenKeys High-tech Materials Co., Ltd.

Functionalized mesoporous silica nanomaterials (FMSN) can be ordered by the physical/structural characteristics of the inert hosting material and/or the chemistries of the functionalization. Both factors control the behavior of the material and its suitability for different tasks and conditions. This ability for optimization and fine-tuning has made FMSN an increasingly promising technology for medical and environmental applications. The most popular configurations have a tendency for large pore volumes but variable pore sizes, high surface areas, and a stability that makes them reliable even in challenging matrixes (low pH, high organic concentrations) and environmental conditions (low–high temperatures) [1–3]. The crystal state of FMSN is shown in Fig. 1. To develop a better understanding of FMSN, it is instructive to consider how they have developed, why, and the technologies that they have superseded.

Research on FMSN has been ongoing for ca. 30 years. In the early 1990s, the initial aim was to expand beyond the confines of zeolite minerals used for ion exchange and contaminant sorption. These hydrated aluminosilicates, comprising of a tetrahedra patchwork of alumina and silica structures, were the most widely used metal/elemental scavengers. Effective as they were, however, they had their limitations, and consequently, there was a demand for new materials (mesoporous silica) with more structured/controlled pores of sizes between 2 and 10 nm. These designs were needed to accommodate molecules that could not be captured by existing zeolites because they were not small enough to fit in the micropores [4]. It was not until 1992 that the first scanning electron microscopy (SEM) micrograph of mesoporous silica appeared, yielding a greater appreciation of their detailed/intricate structure and how synthesis conditions governed morphogenesis [5]. In 1998, Stucky and colleagues synthesized mesoporous silica with larger pores (30 nm), further expanding the range of molecules accessible to the interiors of the Si crystals [6] and opening up new opportunities for functionalization.

It is also worth noting that FMSN have benefited from the increase of interest in nanotechnologies. In accordance with the International Organization for Standardization definition in ISO/TS 80004, nanomaterials encompass not just materials with any external dimension between 1 and 100 nm but those “having internal structure or surface structure in the nanoscale” [7]. With the development of nanomaterials, researchers have discovered the potential of functionalized silica as an advanced chemical material [8, 9]. Combining an ease of surface modification and synthesis, good biocompatibility and stability FMSN quickly became a viable replacement/alternative to zeolites [10].

The other metal-removing agent traditionally used is activated carbon (AC). It has been successfully used in many industrial applications. AC has been applied successfully before to remove As, Cr, and pharmaceutical compounds from water [11–13]. It is also applied for greenhouse gas capture [14], electrical energy applications (as an ultra-capacitor) [15–17], and as an air pollution mitigator [18, 19].

The adsorption capacity traits associated with AC depend principally on their porosity and surface area [20]. Two traditional methods are used to prepare AC that delineate by either physical or chemical activation. Physical activation involves carbonization of organic matter, and then, the resulting char is modified in the presence of an activator (such as CO_2_ or steam). In chemical activation, the activating agents (such as K_2_CO_3_) is used directly for raw material impregnation. Next, heat treatment is applied in an inert atmosphere (various/high temperatures) [20]. Generally, compared with physically activated AC, chemically activated AC has a larger surface area and a smaller pore size, with a wider range of applications [21].

Activated carbon in general has a high internal surface area due to its special sponge-like structure. Moreover, these adsorbents have reasonable chemical stability [22]. Suitable natural ingredients are continually being sought to make low-cost AC materials. To date, AC has been formed successfully from agricultural wastes as diverse as apricot and bagasse, grape seeds and cherry stones, rice husks, coconut, tomato processing solid waste, etc. [12], which highlights its versatility.

The main benefit associated with AC as a clean-up technology for use in wastewater is its overall chemical flexibility and universality. This stems from its large specific surface area, adsorption capacity, wetting characteristics, and heterogeneous porous structure; all of this can be tailored via regulation of the physical/chemical activation steps [23]. The general adsorption capacity of AC depends principally on physical entrainment and then secondly by chemical properties. However, for metal adsorption specifically, the surface chemistry and configuration of outer ligands on the AC are of more importance, providing some element/species selectivity [24, 25]. The problem, though, is that the distribution of functional groups can also change the structure of the AC [24, 25]. This complicates attempts to further functionalize the char, as chemical changes can similarly cause physical modification, with potentially behavior-altering changes for the metal scavenger. Ideally, an inert/stable host is required for functionalization, as this enables better control of the specific chemistries, which is the key to superior performance. Functionalized Si meets these requirements, and this has been partly the catalyst for the rapid development of FMSN.

Many popular metal clean-up methods rely on materials that utilize ion exchange as the main binding mechanism. Here, the process centers on the exchange of ions between a solid and an associated liquid phase [26]. The solid phase is called the ion exchange resin/ion exchanger, because it carries exchangeable ions, insoluble in the liquid phase and during chemical reactions/interactions maintains its structural integrity [27]. The most useful ion exchangers are reversible enabling the materials to be reused multiple times, and based on the charge characteristics of the ion reactions, the materials can be further defined as either cation or anion exchangers [27]. High-quality ion exchangers should have chemical and physical stability, large surface areas, and large ion exchange capacities, and exhibit a relatively fast ion exchange speed [28].

FMSN structural/chemical attributes make them highly attractive to be configured as ion exchangers. However, specialist ion exchange membranes remain the most popular and widely used technology platform for ion exchange-mediated metal removal. This is despite many studies having shown that membrane-based ion exchangers have numerous practical limitations. The most significant of these being poor chemical stability in alkaline environments [29]. For example, in matrixes with a high pH, hydrogen peroxide (including its free radicals) is particularly reactive/damaging for ion exchange membrane modules, making them prone to premature failure [29, 30]. An exception to this is perfluorinated anion exchange membranes, which have outstanding proton conductivity and chemical stability, but suffer from high production costs and a low ion selectivity [30]. In addition, the degradation treatment of waste after the use of ion exchange membranes is still very complicated [31]. As although the elemental target is removed from the matrix, the counter ion still replaces it and this can cause new/secondary problems. In contrast, functional silicas have a more stable chemistry and are less expensive to manufacture. There is also no requirement for a chemical “swap” or exchange to occur, which improves the quality of the treated liquid phase. It is these practical considerations with ion exchange membranes being generally limited by relatively high capital and operating costs, as well as technical or economic constraints [32], that have seen many industries moving away from these systems. Functionalised silica on the other hand have a low operating cost and can be optimized to work in environments with high dissolved organic carbon and low pH [33].

**Fig. 2:**
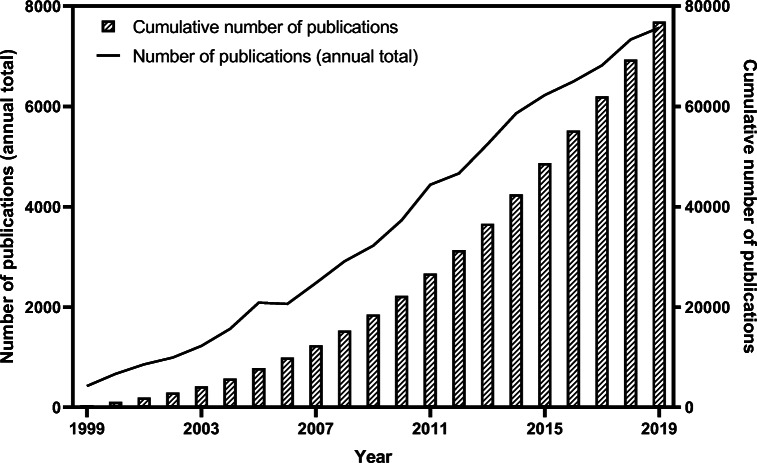
The cumulative number of published papers featuring SBA-15 and MCM-41 FMSN (Data source: Scopus)

This major increase in the popularity of FMSN research is exemplified by two FMSN in particular: Mobile Crystal Corporation (MCM-41) and Santa Barbara Amorphous (SBA-15). These two categories of mesoporous silica have received the most attention. Figure 2 shows that the number of FMSN studies published started to increase rapidly from the early 2000s onwards. Today, there are over 70,000 published papers featuring SBA-15 and MCM-41.

The majority of FMSN studies over the last two decades have focused on the synthesis and preparation of new materials [34–39]. During this period, there has been a constant development of novel structures and functionalizations, with major gains in the understanding of the importance of the inner fine structure of mesoporus Si along with the mechanisms of FMSN binding [40–42]. Depending on the pore length/size, the mass transfer and rate of molecular diffusion are modulated [43, 44]. However, the pore sizes do not need to be uniform through the Si either, and this creates additional scope for tailoring the chemistries for a specific environment or need. For example, Peng et al. [45] were able to create specific mesopore channel regions within the Si with larger surface areas and pore diameters (3.0 to 7.3 nm) by adding 1,3,5-trimethyl benzene alongside cetyltrimethyl ammonium bromide (CTAB) during synthesis. This tuneable micelle core-swelling agent provided a template that controlled the shell architecture of the internal cavities, one that could be easily removed from the silica structure prior to use. It was this manipulation that created the 2D hexagonal mesostructure, with smaller pores in the shell and larger spaces in the silica core, providing the material with its unique, 2-step adsorption-desorption characteristics [45].

However, despite the increasing role and complexity of mesoporous structure as a determinant of adsorption behavior, it is still the functionalization process that dominates the materials’ final characteristics. Thus, introducing different functional groups ultimately controls the metal ions’ adsorption both externally and internally within the adsorbent. For example, carboxylate, sulfonate, and phosphate groups promote the adsorption of metal ions through ion exchange, while amine moieties typically operate by complexation. The adsorption of metals by FMSN in water is mainly based on the use of surface-grafted functional groups, such as amino or mercapto, for complex coordination, thereby achieving selective adsorption of metal ions [46]. Studies have also shown that the adsorption capacity of FMSN increases with an increase in the total concentration of functional groups. The surface area of the adsorbent is obviously an important factor determining functional group concentration [47]. However, the branching structures of the functionalizations, and how many terminal groups there are, also play a defining role.

For the adsorption of nonionic organic matter in water, generally, grafting alkanes onto the FMSN increases surface hydrophobicity, which is beneficial to the capture of organic matter. For the adsorption of ionic organics, the charge density of the solid surface is the key factor for adsorption. The degree of interaction between ionic organics and FMSN changes due to variation in solution pH, because the surface charge of the FMSN and the ionic radius of the ion target are controlled by pH. The ionic strength and ionic composition of the solution also affect the adsorption of ionic organics, especially when inorganic ions compete with organic ions for binding sites [46]. With greater appreciation of the unique adsorbing ability of functional silica, this has prompted more studies to consider a broader range of chemical targets, further expanding the utility of FMSN [48–50]. For example, there are specialist FMSN scavengers for both Hg [51] and Cu [52, 53]. Despite the vast number of wastewater treatment and environmental protection technologies available, FMSN have proven themselves to be a sector leader for metal-chemical removal [54].

### Synthesis of FMSN

There are three main steps in the production of FMSN, synthesis, surface modification, and stabilization. The synthesis of mesoporous silica is commonly performed by base or acid hydrolysis, using an alkoxysilane precursor. The ordered architecture of pores and channels is obtained from amphiphilic micelles which are trapped under the silica network. This template/mold is then removed by either chemical extraction or heat-assisted treatments. The pore length and diameter can be adjusted by altering the amount and type of templating agent used and the reaction conditions [10]. Protective etching with NaOH on silica coated with PVP molecules can be used to form FMSN, especially for crystalline silica such as quartz, cristobalite, and tridymite [55]. Variation in the hollow silica channels is achieved with different etching speeds and durations, and the targeting of outer or interior regions of the particle. However, fine control of pore structure is difficult, with this method approach being more suited to disordered mesopore synthesis [56].

Post-synthesis surface modification and direct synthesis (one-step synthesis/co-condensation) methods are applied to fix organic functionalities to mesoporous silica [57]. For example, in the post-synthesis surface modification of reactive nano-silica, sodium silicate undergoes a hydrolysis reaction as pH decreases. This then generates Si(OH)_4_ monomers which are then developed through a series of dehydration condensation reactions, resulting in a high-density surface functionalization with active hydroxyl groups. At the same time, methoxyl groups of the surface modifier 3-mercaptopropyl-trimethoxysilane (MPTS) then hydrolyze under alkaline conditions to produce the active surhydroxyl group. These active hydroxyl groups generated by MPTS then react with the surface functionalized hydroxyl groups just generated on the silica, forming Si-O-Si bonds and thus providing the mesoporous silica with surface adhered sulfhydryl groups [58].

Through the co-condensation of precursor inorganic framework materials with terminal trialkoxyorganosilanes (such as (R′O)_3_SiR), the mesoporous silica phase can be prepared. This results in organic moieties being covalently anchored to the pore/channel walls of the silica in an orientation that enables them to project directly into the pore walls [57]. The advantage to this functionalization approach is that obstructions within the pores themselves caused by the organic units are minimized. An additional merit is that the modifications are more homogenously distributed within the pores, compared with other techniques. However, the degree of mesoscopic order of the FMSN needs to be monitored. As the ratio between the organic and silica increases in the reaction mixture, a more disordered and less reproducible material is produced [57]. These restrictions though can be conquered with the inclusion of structure-directing agents, producing a class of materials termed periodic mesoporous organosilicas (PMOs). Here, the functionalizations are integral components of the silica structure, resulting in a narrow pore radius distribution and well-organized pore network [57].

The final production stage of FMSN is stabilization. Here, functionalization can be used to give different regions within the Si particle different properties. For example, the core can be made hydrophobic and the shell hydrophilic [59]. This is a key part in improving hydrothermal stability. Hydrophobicity enhances the hydrothermal stability of materials in the aqueous environment, and hydrophilicity ensures biocompatibility. The free silanol (Si-OH) groups on the materials can provide a certain degree of hydrophilicity; with the help of condensers, these will be further used to modify some functional groups, biomolecules, or drug molecules [60, 61].

### Diversity of FMSN’s Functional Groups

Many studies have reported that FMSN with different functional groups have specific selective adsorption functions. In Georgescu’s study [62], synthesized FMSN (TAC-functionalized silica) used for the removal cadmium and copper from water, adsorption results show that 0.1 g of FMSN can extract 94% of Cd(II) and 98% of Cu(II) from aqueous solutions (pH 6). Li et al. [33], through a one-step method, synthesized LMS-AP-FMSN. In a subsequent wastewater treatment experiment, LMS-AP has good metal removal efficiency, and the removal rate of Al, Pb, and Zn reached more than 80%. But the adsorption of Cr from the same waste water was only ca. 70%, while the removal of As was less still at only 30–40% [33]. The huge variety of mesoporous structures, pore network structures, combined with functionalization configurations makes FMSN a highly versatile yet extremely powerful chemical solution to toxic element containment. However, with the enormity of different FMSN configurations, selecting the right materials for the correct conditions is still challenging, and their effectiveness requires case-by-case validation. In the following sections, the application of FMSN in tackling environmental pollution will be discussed.

## Current Applications and Future Directions of FMSN

### The Use of FMSN in Water and Food Security

FMSN are currently widely used to absorb numerous metal ions and compounds from aqueous solutions [63–66] (Table 1). For example, Xia et al. [63] synthesized a variety of different FMSN to study comparatively, FMSN adsorption capacity for TTEs in water. In their absorption experiments, Cd(II), Mn(II), Pb(II), and Fe(III) concentrations were all ~ 0.5 mg L^−1^ in drinking water with a pH of ~ 4; it was found among the types of FMSN tested; S16-1N completely removed these four elemental impurities (< detection limit). The excellent performance of S16-1N can be ascribed to its high pore volume and specific surface area, along with possessing high accessibility to its functional group ligands [63]. Chatterjee et al. [66], by using a modified Stober process, synthesized cubic mesoporous silica, then it was functionalized to produce a fluorogenic silica probe material (SiO_2_@AZOL). The adsorption experiment of the fluorogenic silica probe material was performed in a solution with a pH of ca. 7. It was found that this material showed an extraction efficiency of around 99% for HSO_3_^−^, Cd(II), Hg(II), Zn(II), and Cu(II), with adsorption capacities of 873, 633, 630, 412, and 260 mg g^−1^, respectively [66]. For existing industrial applications, in Guizhou, China, the FMSN iMoLboX has been successfully applied to retrieve precious metals in addition to the removal of TTEs from a variety of process waste streams. For example, iMoLboX has achieved a 96.14% removal efficiency of As in medium/strong acid solution (mass fraction of 65–85%) containing 300 μg L^−1^ As. While it possesses a removal efficiency for Cu of 92.97% in aqueous solutions containing 30 mg L^−1^ CuSO_4_ (Fig. 1). In addition, iMoLboX FMSN can effectively extract Rh from acidic aqueous solutions containing 57 mg L^−1^ Rh(III), and remove Pd from organic alcohol solutions containing 562 mg L^−1^ Pd(II); in both cases, removal efficiency is > 99% [69]. But the adsorption efficiency of ion exchange resins for Rh(III) and Pd(II) from 8.9 mg L^−1^ Rh(III) solution and 43.9 mg L^−1^ Pd(II) solution is only about 90% [70], while the removal ratio of AC for Pd(II) from 100 mg L^−1^ Pd(II) solution is 93.5% [71].

**Table 1:** A summary of FMSN applications used to remove metals and compounds from waters

MFS	Species	Dominant system/scheme	Key finding	Ref.
SBA-15	Cu(II ) and Cd (II)	The adsorption of copper and cadmium ions was studied	The adsorption capacity of SBA-15 for Cu (II) and Cd (II) in a single metal solution was determined and related to the density and structure of the organic groups grafted on the surface of SBA-15.	Georgescu et al. [62]
SBA-15	Pb(II) and Cu(II)	Adsorption experiment	The adsorption of thiol-group SBA-15 and The amino-group SBA-15 to Cu(II) and Pb(II) was studied.	Lee et al. [67]
SBA-15	U(VI)	Adsorption experiment	SBA-15-PA showed not only a good sorption ability and a desirable selectivity for U(VI) over a range of competing metal ions but also an excellent reusability.	Wang et al. [68]
Porous silica materials S8, S12, S16, and SBA	Cd(II), Pb(II), Fe(III), Mn(II)	Heavy metal adsorption experiments	Monoamino-functionalized silica S16-1N shows effectively remove heavy metal Cd(II), Pb(II), Fe(III), and Mn(II).	Xia et al. [63]
Mesoporous silicas MCM-41 and MSU-H	Hydrogen peroxide	Hydrogen peroxide adsorption analysis	MSU-H silica have better absorption ability than MCM-41 because its lager pore size.	Lewandowski et al. [64]
LMS-AP	Pb (II)	Pb(II) adsorption experiments	In actual industrial wastewater treatment process, LMS-AP had a better Pb(II), Zn(II), and Cr (VI) removal efficiency of 80% and As (V) of 30–40% removal efficiency at initial pH 4.	Li et al. [33]
Fe_3_O_4_ @SiO_2_ -NH_2_&F13	Perfluorinated compounds (PFC)	Sorption experiment	For the removal of PFC from surface water samples, Fe3O4 @SiO2 -NH2&F13 has a good anti-interference ability, and shows good removal efficiency (86.29%) for the nine PFCs analyzed in this study, as well as its reusability and stability.	Zhou et al. [65]
SiO2@AZOL	Hg(II), Cd(II), Cu(II), and Zn(II)	Adsorption studies	The material shows a high adsorption capacity for a variety of toxic metal ions (Hg(II), Cd(II), Cu(II), and Zn(II)).	Chatterjee et al. [66]

Fluorine is highly reactive and forms a great variety of chemical compounds. It is used extensively in the nuclear and metal processing industries, in electronics, as a surfactant, features prominently in polymers and in agrochemicals where it features within pesticide formulations. Fluorinated compounds though are major environmental pollutants and can be difficult to remove from waters. Zhou et al. [65] developed a novel silica functionalized nanocomposite (Fe_3_O_4_@SiO_2_-NH_2_&F_13_); this material showed a good selectivity for perfluorinated compounds (PFCs). In a 1-L water sample fortified with PFC concentrations ranging from 0.5 to 50 ng L^−1^, the composite possessed a much better removal efficiency (86.29%) for PFCs than that (58.61%) of powdered AC [65].

FMSN can also be used to extract drug residues from food [72]. In this example, three different mesostructured silicas (SBA-15-C18, MSU-2-C18, HMS-C18) with added octadecylsilane groups were trialed. Despite the functionalization approach being identical, the different silica structures evolved distinctive binding properties. SBA-15-C18 was the least effective scavenger due to hydrophobic and polar secondary/hydrogen bonding interactions caused from the functionalization leaving a high frequency of non-modified silanol groups in the silica. However, HMS-C18 proved a promising multi-drug sorbent. Out of the panel of 26 veterinary drug residuals tested from bovine meat samples, the extraction efficiency for more than half of these compounds was over 80% [72]. Although the direct use of FMSN in food safety is new, the successes to date provide further evidence of the materials’ wide-ranging applications, across diverse chemical conditions. However, perhaps, it is the use of FMSN to combat soil pollution that might be the materials’ greatest contribution to our future food safety [73].

### The Use of FMSN in Soil Research

FMSN has good compatibility for use within soil because it is inert, highly stable, and soils naturally already contain high proportions of silica. The application of FMSN in soil research is a theme that has only emerged relatively recently. The two principle directions being soil remediation (direct amendment) [73] and in situ measurement [74].

Although the use of functionalized silica in soils is relatively new, there have already been a number of successful applications. For example, diacetylmonoxime-functionalized silica gel has been used to remove Cu from fly ash-ameliorated soil samples [75]. In this study through FTIR, element analysis, BET surface area analysis, and ^13^C CPMAS NMR spectroscopy, it was shown that the FMSN have high selectively for Cu removal with a preconcentration factor of 250; sorption capacity reached 0.93 mmol g^−1^. Kinetic parameters and isotherms correlated well with the pseudo-second-order and Langmuir models [75]. Another important study reported that FMSN can limit the absorption of toxic metals and As by plants through reducing bioavailability. In this example, a soil with an As concentration of 9.87 ± 1.22 mg kg^−1^ had the fraction of unavailable As increased from 40 to 77% after FMSN was applied [76]. Similarly, Lian et al. [58] used FMSN in contaminated soil to selectively immobilize Pb and Cd. Results showed that the FMSN applications can decrease the pools of bioavailable Cd and Pb from 12 mg kg^−1^ and 1194 mg kg^−1^ to 0.2 mg kg^−1^ and 10 mg kg^−1^, respectively, with the immobilization efficiency of Pb and Cd reaching 99.12% and 98.23%. More importantly, this FMSN exhibited a low effect on other soil parameters. While the Cd and Pb species immobilized by FMSN exhibited greatly increased acid resistance [58], an attribute making FMSN suited for long-term remediation roles.

Recently, a series of studies has shown that FMSN can be used for remediating Cd-contaminated agriculture soil [73, 77]. The stabilization efficiency of Cd by FMSN reached up to 91.2% with just a 1% application. At the same time, the migration rate of Cd in the soil decreased from 45.8 to 19.0%, and the effect of the addition of FMSN on the soil properties was negligible [77]. In addition, FMSN reduced the leaching rate of Cd in the soil (36.0%) and bioavailability (54.3%); the concentration of Cd in crops decreased more than 50% [73].

Directly, addressing pollutant bioavailability with amendments is one solution to improve soil health. However, any implementation of such technologies needs sufficient knowledge of in situ conditions and subsequent pollutant mobility. Over the past couple of decades, the passive sampling technology of DGT has emerged as a leading method for the measurement of bioavailability for inorganic and organic contaminants in soil. However, the connection with FMSN is that they are increasingly being used as DGT substrates, opening up a range of exciting avenues in soil testing, new rapid analysis approaches, and for high-resolution 2D ion-mapping of pore water chemistries.

## The Use of FMSN in DGT Research

### DGT Introduction

DGT was originally developed for aquatic systems, then evolved into an established technique to understand chemical lability and speciation in soil [78]. Comprising of a base unit, a cap, and thin film stack system (consisting of a membrane filter, diffusive gel, and binding layer), the DGT configuration is robust, easy to work with in the environment, and crucially, units do not require individual calibration [78]. The binding layer provides the device’s selectively, while the diffusive layer controls solute transport and defines the intensity of the metal flux induced by the DGT device. Together, they both determine the measurement characteristics of a specific/individual DGT configuration.

In practice, DGT devices are easy to deploy in water/sediment/soil; for a detailed review of the technique, refer to Davison [78]. After the DGT binding layer is recovered and eluted, quantitative analysis of the eluents can be achieved through a range of commonly available analytical techniques, including inductively coupled plasma mass spectrometry (ICP-MS), inductively coupled plasma optical emission spectrometry (ICP-OES), atomic absorption spectroscopy (AAS), and X-ray fluorescence (XRF). Environmental samples are prone to a wide range of measurement interferences due to matrix complexity. A further benefit of the DGT approach is that there is a preconcentration of the target analyte. In the following sections, the use of DGT and FMSN for the measurement of TTEs in soil will be discussed from the perspective of Hg and As pollution.

### Hg In Situ Sampling by DGT

Mercury (Hg) pollution remains a key pollution focus, not only is it a potent human poison, but it can cause widespread environmental damage [16, 17]. Mercury exposure can arise naturally from rock weathering, volcanic activity, and geological deposition [79]. However, the main sources of Hg release are from human activities, particularly waste incinerators, residential coal for heating and cooking, coal-fired power plants, and mining for gold and other metals [16, 17, 79, 80]. The fate and behavior of Hg are difficult to predict, which complicates the assessment of toxicological risk, due to the wide range of species and complexes found in the environment. Elemental mercury (Hg0), referred to as quick silver, is the most volatile species, forming vapor easily at typical ambient temperature/pressure [81]. Hg(OH)_2_, Hg(OH)Cl, and HgCl_2_ are common inorganic mercury (iHg) compounds in freshwaters. However, because the Cl concentration in seawaters is higher, Hg speciation trends are also typified by HgCl^+^, HgCl_2_, HgCl_3_^−^, and HgCl_4_^2−^. Methylmercury (MeHg) is an organometallic Hg species, characterized by extreme toxicity, making it the critical contaminant/pollutant in the environment [81].

Soil is an important reservoir of anthropogenic Hg release, playing a key role in the Hg cycle [82]. The soil environment is a heterogenous micro-landscape of solid-solution-gas interfaces, and fluxes between these compartments are dominated by the soil phase characteristic/composition, where partitioning of the Hg is concentrated. Mechanistically, the kinetics of Hg mobility can be broadly segregated into two groupings. The first is an immediate/quick reaction scheme forming outer-sphere complexes involving cation exchange, with a multitude of different ligand types, both organic and inorganic. The other group involves the formation of stable colloid complexes that diffuse within the interior of the soil particles. The bonds formed are inner sphere and recalcitrant, characterized by slow release. However, these stores should not be considered as insignificant as they can provide a long-term and sustained source of bioavailable Hg [83, 84].

DGT has been shown to be effective at predicting Hg uptake in plants [85]. It acts to introduce a controlled perturbation into the soil system—replicating the processes evoked by the plant root. The method is also advantageous for in situ measurement of Hg speciation, because it preserves the stability and distribution of the Hg species, while also preconcentrating the target analyte during sampling [86]. DGT and HPLC-ICP-MS are appropriate tools for the evaluation of rhizosphere Hg bioavailability in estimating the risk from contaminated soils [84]. Liu et al. [85] predicted MeHg uptake by rice plants (*Oryza sativa* L.) by using the DGT technique to detect bioavailable MeHg in pore water; the DGT probe used a 3-mercaptophyl-FMSN. A significant positive correlation between MeHg flux in soil measured by DGT and MeHg flux in rice roots (*R* = 0.853, *p* < 0.01) was observed. The correlation between these two flux parameters indicates that DGT can provide a quantitative description of the rate of update of this bioavailable MeHg, and that the DGT can predict the bioavailability of MeHg in rice paddy soil [85]. Ridošková et al. [87] used Ambersep GT74 and Duolite GT73 gels as DGT binding layer for measuring the bioavailability of Hg in contaminated soils. The DGT experiments indicated that Duolite GT73 shows similar Hg accumulation properties as Ambersep GT74; the maximum Hg accumulation for these two resin gels was obtained after 12 days of deployment in the soil, about 27 and 33 ng/disk, respectively. In four different soil deployment experiments, the range of DGT available Hg compared with total Hg ranged between 2 and 10% [87]. Huu Nguyen et al. [88] measured the bioavailability of Hg in soil for the earthworm *Eisenia Fetida* by using DGT which used FMSN (3-mercaptopropyl-functionalized silica) as the binding phase. Soils used in Hg exposure experiments were prepared with different pH (4.6, 5.6, and 6.2) and varying peat moss concentrations of 5, 10, 15, and 20%. DGT was deployed in the soil with approximately 35–40 earthworms and removed across a series of timepoints spanning half-a-day through to 10 days. The calculated uptake efficiency for Hg for this binding gel was 91 ± 3.4%; the DGT was able to predict the Hg concentration of earthworm tissue effectively, unlike comparative measurements of pore water/acid-extractable Hg [88]. It can be seen that DGT is a dependable technique that can be used to measure the bioavailability of Hg in soil. Compared with other DGT, DGT using FMSN as the binding phase has better absorption efficiency for Hg. For more research papers about Hg in situ sampling by DGT, see Table 2.

**Table 2:** A summary of FMSN and non-FMSN DGT configurations used for in situ sampling of Hg

Binding phase	Species	Matrix	Dominant system/scheme	Key finding	Ref.
Chelex-100 and Spheron-Thiol	MeHg, CH3CH2Hg+, Hg(II)	Soil	HPLC-ICP-MS, DGT	DGT techniques is a suitable tool for the estimation of Hg root availability and in assessing the risk from contaminated soils.	Cattani et al. [84]
Duolite GT73 and Ambersep GT74 gel	Hg(II)	Water	DGT	The agarose gel was found as a diffusion gel for mercury measurement in DGT techinique.	Docekalová and Diviš [89]
3-mercaptopropyl-functionalized silica (Sigma-Aldrich)	Hg(II), MeHg	Soil	DGT	A new resin gel Ambersep GT74 was used in DGT technology, which showed similar Hg accumulation characteristics to Duolite GT73.	Ridošková et al. [87]
3-mercaptopropyl-functionalized silica (Sigma-Aldrich)	MeHg	Soil	DGT	Research shows that DGT can predict bioavailability of MeHg in paddy soil.	Liu et al. [85]
3-mercaptopropyl-functionalized silica (Sigma-Aldrich)	Total Hg	Soil	DGT	DGT-measured Hg flux is a better tool than conventional methods for predicting Hg bioavailability for earthworms inhabiting diverse types of soil.	Huu Nguyen et al. [88]
3-mercaptopropyl-functionalized silica (Sigma-Aldrich)	Total Hg	Soil	DGT	The effectiveness of DGT technology in predicting plant uptake of mercury was confirmed.	Turull et al. [90]
3-mercaptopropyl-functionalized silica (Sigma-Aldrich)	Total Hg, MeHg	Water	DGT	Hg speciation in aquatic systems was investaged, found that Labile MeHg in the sediment of the Gulf represents up to 75% of total labile Hg.	Bratkič et al. [91]

### In Situ Sampling of As with DGT

Arsenic is the 20th most abundant element in the earth’s crust and can be present in the environment in four oxidation states As(-III), As(0), As(III), and As(V) and as both organic and inorganic species [92]. The general order of toxicity of the different arsenic species is as follows: arsenite > arsenate > monomethyl arsenate (MMA) > dimethyl arsenate (DMA) [92, 93]. While, the total content of As in the environment is largely decided by the degree of environmental pollution and associated geology. Localized redox conditions play a main role, along with the microbiome in controlling the concentrations of As(III) and As(V) [92], while methylation/demethylation is subject to biotic controls Su et al. [94]. This creates a diverse range of As chemistries in situ that can be difficult to capture.

In 2012, Bennett et al. performed selective measurement of total inorganic arsenic and As(III) using separate DGT sampler units configured with different binders. Here, it was determined that As(III) was the main substance transferred from the sediment (solid phase) to pore water. They also showed that the DGT technology can be used under a range of deployment conditions to investigate arsenic morphology and its migration potential [95]. Wang et al. [96] used Zr-O DGT to systematically study the concentration variation of unstable As in the sediments of Hongze Lake; the recovery of Zr oxide DGT was 88%. It was found that the concentration of labile As in the sediment profiles varied considerably (ranging from 0.15 to 4.15 μg L^−1^) [96]. Gorny et al. [97] used a Zn-ferrite (ZnFe_2_O_4_) substrate as the DGT-binding phase to measure total arsenic in river water and sediment pore water. Results shown ZnFe_2_O_4_ have a better sorption capacity for As(III) compared with ferrihydrite and Metsorb binding gels, with the sorption capacity of ZnFe_2_O_4_ reaching 54,000 ng for As(III) [97]. In 2011, Bennett et al. first used FMSN as a DGT-binding substrate to selectively measure As(III), and its absorption efficiency for As(III) was > 99%, validating the feasibility of this method. The reason why mercaptopropyl-silica have highly selective adsorption ability for As(III) is because of the strong complexation of H_3_AsO_3_ by thiol (S-H) groups [98]. Gorny et al. [99] also reported on the use of FMSN as the DGT-binding layer and found that the use of matrix corrected elution factors to rectify the interaction with sulfides can ameliorate the measurement of As(III) in 3-mercapto-silica (3MP)-binding gels; the concentration of As(III) recovered from the 3MP-DGT samplers reached 103 ± 7.0 and 93.8 ± 8.6% (mean ± standard deviation) of the initial As(III) concentration in seawater and freshwater, respectively [99] (Table 3).

**Table 3:** A summary of FMSN and non-FMSN DGT configurations used for in situ sampling of As

Binding phase	Species	Matrix	Dominant system/scheme	Key finding	Ref.
Metsorb and mercapto-silica	Total inorganic arsenic and As(III)	Water and sediment	DGT, DET, ICP-MS	Demonstrated the capabilities of the DGT and DET techniques for investigating arsenic speciation and mobilization over a range of sediment conditions.	Bennett et al. [95]
Zirconium (Zr) oxide and 3-mercaptopropyl-functionalized silica	Total As	Sediment	DGT, atomic fluorescence spectrometry (AFS)	Prove DGT is a reliable and high-resolution technique that can be used for in situ monitoring of the labile fractions of As and Hg in sediment in fresh water bodies.	Wang et al. [96]
Ferrihydrite (Fe) and titanium dioxide (Ti)	As(III)	Water	DGT, DET, ICP-MS	The concentration of arsenic in the pore water of contaminated rice fields was measured, and the importance of pore water in the measurement of arsenic was emphasized.	Garnier et al. [100]
Zn-ferrite (ZnFe2O4) and 3-mercaptopropyl-functionalized silica	Total As and As(III)	Sediment	DGT, ICP-MS	The new Zn-ferrite binding gel is well adapted for determining total As concentrations, and phosphate interference on As measurements has been demonstrated whatever the binding gel.	Gorny et al. [97]
3-mercaptopropyl-functionalized silica and Metsorb	Total inorganic arsenic and As(III)	Water	DGT, ICP-MS	First used 3-mercaptopropyl-functionalized silica as DGT binding phase to selectively measure As (III), and proved the feasibility of this method.	Bennett et al. [98]
3-mercaptopropyl-functionalized silica, ferrihydrite, Metsorb, zinc ferrite, and zirconium dioxide	Total As and As(III)	Sediment	DGT, ICP-MS	Comparison of the adsorption capacity of different binding gels and found that the use of unconventional elution factors to correct the interaction with sulfides can improve the determination of As (III) in 3-mercaptopropyl-functionalized silica binding gels.	Gorny et al. [99]

### DGT-Two-Dimensional Chemical Imaging

**Fig. 3:**
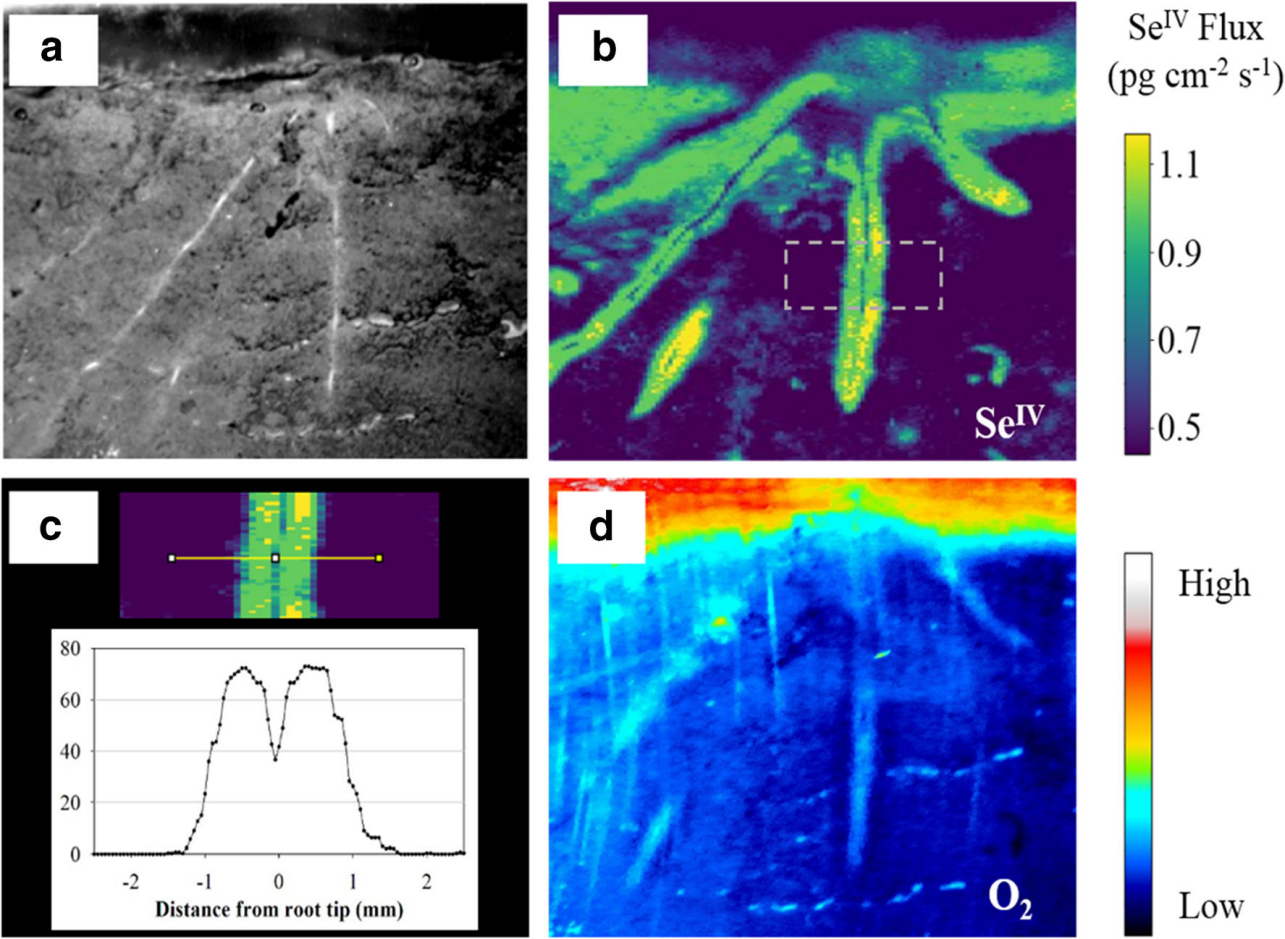
Two-dimensional representation of Se^IV^ and O_2_ around a set of rice root. **a** Photograph of rice root grown in Se-contaminated soil. **b** Visualization of Se^IV^ around a set of rice roots. The outlined position of root that is featured in (**c**) is indicated by gray dash markings. **c** Se^IV^ species in the soil solution with distance from the root zone. **d** Oxygen distribution imaged by an O2 planar optode sensor [103]. Reprinted with permission from Shi X, Fang W, Tang N, Williams P, Hu X, Liu Z, Yin D, Ma L, and Luo J. In situ selective measurement of Se(IV) in waters and soils: diffusive gradients in thin-films with bi-functionalized silica nanoparticles. Environmental Science & Technology. 2018;52(24):14140-14148.). Copyright (2020) American Chemical Society

The acquisition of environmental/soil measurements in the majority of studies to date prioritizes the sampling of individual biogeochemical compartments. Far less consideration is given to the speciation, transformation, and exchange of elements across interfaces between different niches/zones, and the processes controlling these events. 2D high-resolution diffusive gradients in thin films (HR-DGT) are a key component of one of the few existing tools that can capture multi-elemental solute dynamics/interactions in appropriate submillimeter scales, to suitably understand/decipher interface fluxes. The usual operation of this chemical imaging approach uses a multi-layered system of DGT and planar optodes to obtain the HR-2D images. This is not intended to be a comprehensive review of this emerging technology; for this, we recommend Santner et al. [101] and Santner and Williams [102]. However, FMSN are increasingly been used in these ion-mapping applications, because of their high capacities, environmental tolerances, and species specificity. For example, Shi et al. [103], through mapping the 2D chemistry of a rice rhizosphere, were able to capture the distribution of Se(IV) and oxygen simultaneously. Here, the DGT substrate used was a bi-functionalized SBA15 FMSN, which provided a selective measurement for Se(IV) (Fig. 3) [103]. What was apparent from these chemical images was that Se(IV) mobility is restricted to the rooting zone and with localized redox conditions controlling release patterns. This is despite the Se amendments having been thoroughly mixed through the soils prior to the addition of the rice plants.

**Fig. 4:**
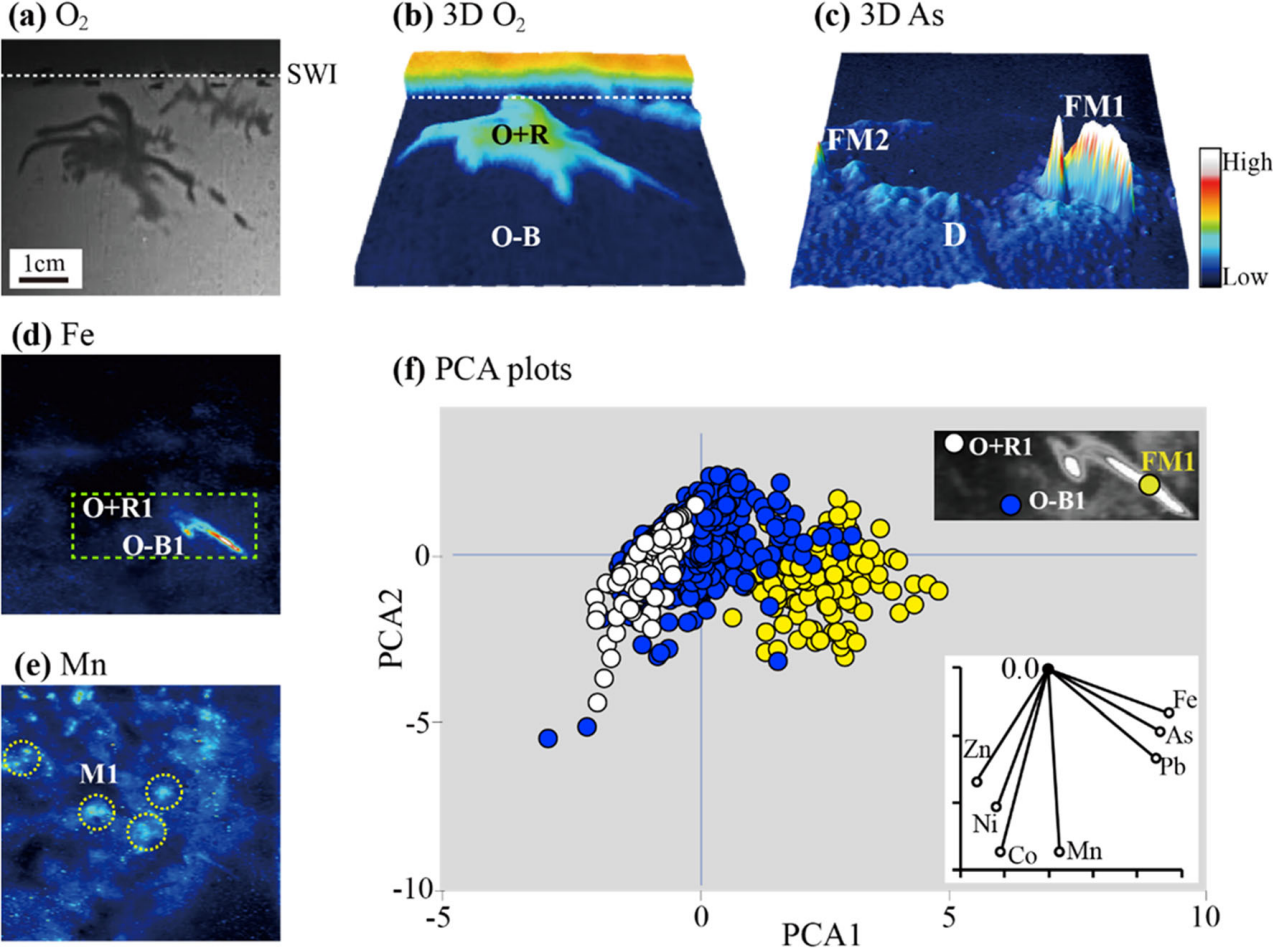
Solute fluxes around a set of four-week-old rice roots with SPR-IDA DGT and O_2_ planar optodes. **a** Image of O_2_ distribution obtained before the deployment of the sandwich sensor. The horizontal-dashed lines show the soil-water interface (SWI). **b** 3D plot of O_2_ distribution in the rice rhizosphere with the sandwich sensor, O+R indicates the aerobic rhizosphere, O-B indicates the anaerobic bulk area. **c** 3D plot of As fluxes in rice rhizosphere with the sandwich sensor. FM indicates flux maxima around the root tip apice. D indicates the flux depletion zones. **d** Fe fluxes in the rhizosphere. The green box shows the corresponding data extraction region/transect used for PCA analysis. **e** Mn fluxes in the rhizosphere. The yellow circles indicate flux microniches (label as M1). **f** PCA plot of elements in different regions, aerobic rhizosphere (O+R), non-rhizosphere/anaerobic soil (O-B), and flux maximal around root tip apice (FM1). For all images, the metal fluxes (*f*_DGT_, pg cm^−2^ s^−1^) and oxygen concentration (percent air saturation) increased sequentially with the color scale shown from blue to white. The scales in the figure represent the following ranges from 0 to 100% for O_2_, from 0.004 to 0.126 for As, from 0 to 42.144 for Fe, and from 0.71 to 22.39 for Mn [104]. Reprinted with permission from Yin D, Fang W, Guan D, Williams P, Moreno-Jimenez E, Gao Y, Zhao F, Ma L, Zhang H, and Luo J. Localized intensification of arsenic release within the emergent rice rhizosphere. Environmental Science & Technology. 2020;54(6):3138-3147.). Copyright (2020) American Chemical Society

**Fig. 5:**
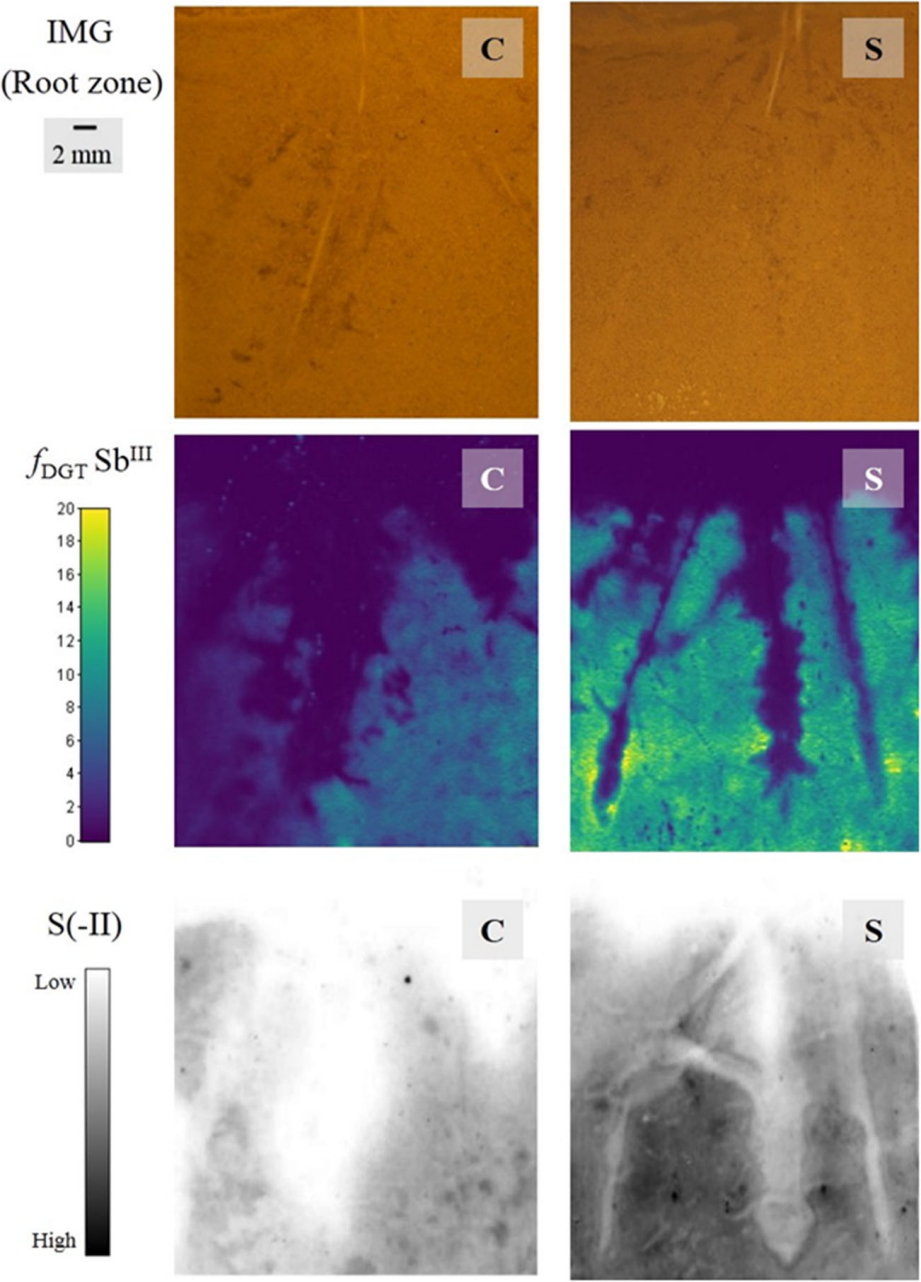
Photograph of rice root after planting in the blank soil (C) and the soil treated with 100 mg kg^−1^ sulfur (S) (top). High-resolution 2D profiles of Sb^III^ and dissolved sulfide in the rhizosphere of rice obtained by MSBA-DGT (middle) and AgI-DGT (bottom) for 24-h deployments, respectively [74]. Reprinted with permission from Fang W, Shi X, Yang D, Hu X, Williams P, Shi B, Liu Z, and Luo J. In situ selective measurement based on diffusive gradients in thin films technique with mercapto-functionalized mesoporous silica for high-resolution imaging of Sb(III) in soil. Analytical Chemistry. 2020;92(5):3581-3588.). Copyright (2020) American Chemical Society

This recent interest in the root-soil interactions within rice rhizospheres is understandable. Rice is so widely cropped and plays a fundamental role in global food security, yet is significantly threatened by trace element imbalances, either deficiencies or over-exposure. The below-ground processes around the roots are very challenging to measure because of the complexity and instability of the environment due to strong zonation in both chemical and biological effects. This means that there is still much to learn about the biogeochemistry of paddy soils and in particular the role of these fine-scale, localized features on plant health and quality. This is demonstrated by the recent DGT-optode imaging of greatly enhanced fluxes of As, Pb, and Fe(II) mobilization immediately adjacent to lateral root tips (Fig. 4) that forms simultaneously within a zone of P depletion and co-currently with a root-induced oxidation front [104, 105]. 2D HR-DGT applications lend themselves very well to this study, being a low-disturbance sampling system that was initially developed for flooded wetland sediments [105]. Following on from this work, Fang et al. [74] expanded the range of species that can be measured in a rice rhizosphere by developing a mercapto-functionalized SBA-15 FMSN DGT for Sb(III) solute imaging. Compared with commercially available 3-mercaptopropyl-functionalized silica, the newly synthesized FMSN was distributed more evenly within the sampler, making it a better candidate material for LA-ICP-MS analysis. Furthermore, the DGT multi-system was also deployed with an AgI gel layer to study the influences of sulfur application on Sb(III) solubility (Fig. 5) [74]. It was found that sulfur additions were contributing to greater release of Sb(III) compared with the non-amended control, and there was a strong spatial trend between Sb(III) and S(-II). There were also hotspots of mobilization that formed around root tip zones, similar to the patterns observed for As and Pb [104, 105].

Traditionally, to extend the range of element/species measurements captured by the DGT/optode systems, binders have been mixed together, or the combination of binder layers has been increased. What is exciting about FMSN approaches is that these properties can be potentially delivered by one binder phase, given the correct functionalization approach, providing a molecular-based solution to expanding and improving chemical imaging delivery.

## Limitations and Considerations for FMSN Use in Soils

As documented in this review, FMSN are a diverse and rapidly developing class of materials with a wide array of different physio-chemical properties. However, there is still a lot that remains unknown about the immediate and persistent effects these incredibly versatile and varied materials can have on soil/plant health. Or what the interplays are when soil type, management practices and climate all interact. Answering some of these questions just involves more testing. However, as a first principle, the toxicity of inorganics in severely polluted soils would have a dominant influence on soil processes and function. Therefore, reducing the mobility of harmful metal(loids) would render a restorative effect. However, this would of course be dependent on specific FMSN, dosage, and the soil environment.

The greatest uncertainly, though, in terms of long-term safety and behavior lies with the smallest of the FMSN technologies. Those Si materials, at the lowest end of the nano-scale spectrum, even when not chemically functionalized, can give rise to unexpected interactions with cellular biological systems/metabolism. Sometimes, these are beneficial. For example, in addition to the widely known antagonisms associated with Si and As(III) uptake in plants. It has recently been observed that SiO_2_ nanoparticles (NPs) provide additional protection to plant cells subject to As stress, mediating a strengthening of plant cell walls by increasing their thickness and altering their composition [106]. Likewise, in maize, field applications of SiO_2_-NPs resulted in a large improvement to growth, but interestingly, there were disruptive effects as well with the Si found to induce a pesticide response for the crop during storage [107]. Similarly, some soil invertebrates demonstrate avoidance behaviors to SiO_2_NP Santos et al. [108], and it has been shown that they can increase soil bacteria community diversity [109].

SiO_2_NP place within the top five commercial nanoproducts globally [108, 110] with applications ranging from paint stabilizers through to food agents [111]. Therefore, extensive material flows of SiO_2_NP into the environment are already ongoing [111]. However, when trying to isolate and understand the already complex mechanisms of FMSN interactions within the plant-soil continuum, it would be prudent to work initially with larger particle size FMSN. Not only would these have more predictable mobility in soils but they would simplify the interpretation of the soil chemical trends, making it easier to match a specific functionalization to its response in situ.

## Outlook/Conclusion

Global soil health continues to be threatened by trace element pollution. This damage is inextricably linked to our continued intensive agri and industrial activities, demand for natural resources, and poor waste management and material recycling/reuse. This land degradation can be broadly viewed as either a general increase in metal loading into soil systems, or modification of existing elemental reservoirs, or a mixture of both processes which results in enhanced bioavailability and toxicity. New technologies are required to counter these pollution challenges, and the behavior and properties of FMSN are highly suitable for this mission. Firstly, Si is already a major constituent of soils, and is highly stable in the environment. It is these traits that provide a cost-effective and resistant platform from which to optimize the functionalization which in combination with the architecture of the pores and channel networks within the Si controls the binding chemistries and their specificities to desired targets. These dual physical and chemical “levers” enable the production of materials with a wide range of behaviors and characteristics that can be controlled very precisely by the developers. Used predominantly to clean waters and “wet” waste discharges, there is exciting potential for more soil applications to reduce toxic metal uptake in crops and as permeable reactive barriers to halt pollutant plume migrations and protect vulnerable groundwaters.

The other aspect of FMSN applications is in their role in environmental measurement. In this review, we have focused on the use of FMSN in the widely used/popular passive sampler DGT, examining this from the perspective of two soil pollutant priorities Hg and As, alongside the emerging new DGT applications of HR-2D ion-mapping. In conclusion, the benefits FMSN in an environmental setting are gaining increasing attention, as more application successes continue to be shown. The versatility of the FMSN platforms is perhaps best illustrated by their large range of uses, fulfilling a research need across the diverse themes of pollutant characterization, analysis, monitoring, prevention, treatment, and remediation. However, there is still much to be discovered about how FMSN perform in different soil environments, especially in wetland agri-ecosystems like rice paddies where inorganic pollutants such as As are of particular concern [105, 112]. It is in these systems where in order to improve air quality, worldwide bans on rice straw burning have resulted in the repurposing of the straw which was once cycled back into the soil. This export removes millions of tonnes of Si. This is of concern because of links Si depletion has on harvest failures, rampant pest/disease outbreaks, and enhanced plant uptake of As [113]. How FMSN could play a role in efforts to resupply Si to these damaged soils is still unresolved, but certainly for the worst metal-impacted sites, its use carries appeal.

## Electronic supplementary material

ESM 1 (DOCX 74 kb)

## References

[1] Weiser D, Boros Z, Nagy J, Hornyánszky G, Bell E, Sátorhelyi P, Poppe L (2017). Chapter 15 SynBiocat: protein purification, immobilization and continuous-flow processes.

[2] Jurado-Gonzalez M, Li Ou D, Sullivan A, Wilson J (2002). Synthesis, characterisation and catalytic activity of porous vanadyl phosphonate-modified silicas. J Mater Chem.

[3] Al-Hashimi M, Roy G, Sullivan A, Wilson J (2005). Selective oxidations of sulfides to sulfoxides using immobilised cerium alkyl phosphonate. Tetrahedron Lett.

[4] Slowing I, Vivero-Escoto J, Trewyn B, Lin V (2010). Mesoporous silica nanoparticles: structural design and applications. J Mater Chem.

[5] Yang H, Ozin G, Kresge C (1998). The role of defects in the formation of mesoporous silica fibers, films, and curved shapes. Adv Mater.

[6] Zhao D (1998). Triblock copolymer syntheses of mesoporous silica with periodic 50 to 300 angstrom pores. Science..

[7] Buzea C, Pacheco I, Robbie K (2007). Nanomaterials and nanoparticles: sources and toxicity. Biointerphases..

[8] Kresge C, Leonowicz M, Roth W, Vartuli J, Beck J (1992). Ordered mesoporous molecular sieves synthesized by a liquid-crystal template mechanism. Nature..

[9] Bibby A, Mercier L (2002). Mercury (II) ion adsorption behavior in thiol-functionalized mesoporous silica microspheres. Chem Mater.

[10] Jadhav S (2014). Incredible pace of research on mesoporous silica nanoparticles. Inorg Chem Front.

[11] Asadullah M, Jahan I, Ahmed M, Adawiyah P, Malek N, Rahman M (2014). Preparation of microporous activated carbon and its modification for arsenic removal from water. J Ind Eng Chem.

[12] Niazi L, Lashanizadegan A, Sharififard H (2018). Chestnut oak shells activated carbon: preparation, characterization and application for Cr (VI) removal from dilute aqueous solutions. J Clean Prod.

[13] Grover D, Zhou J, Frickers P, Readman J (2011). Improved removal of estrogenic and pharmaceutical compounds in sewage effluent by full scale granular activated carbon: impact on receiving river water. J Hazard Mater.

[14] Sawant S, Munusamy K, Somani R, John M, Newalkar B, Bajaj H (2017). Precursor suitability and pilot scale production of super activated carbon for greenhouse gas adsorption and fuel gas storage. Chem Eng J.

[15] Arena N, Lee J, Clift R (2016). Life cycle assessment of activated carbon production from coconut shells. J Clean Prod.

[16] Kim M, Kim K, Park S, Roh K. Hierarchically structured activated carbon for ultracapacitors. Scientific Reports. 2016a;6(1). 10.1038/srep21182 PMC4754731 26878820

[17] Kim K, Kabir E, Jahan S (2016). A review on the distribution of Hg in the environment and its human health impacts. J Hazard Mater.

[18] Rashidi N, Yusup S (2017). Potential of palm kernel shell as activated carbon precursors through single stage activation technique for carbon dioxide adsorption. J Clean Prod.

[19] Mohamad Nor N, Lau L, Lee K, Mohamed A (2013). Synthesis of activated carbon from lignocellulosic biomass and its applications in air pollution control—a review. J Environ Chem Eng.

[20] Yagmur E, Ozmak M, Aktas Z (2008). A novel method for production of activated carbon from waste tea by chemical activation with microwave energy. Fuel..

[21] Ahmed M, Hasan Johir M, Zhou J, Ngo H, Nghiem L, Richardson C, Moni M, Bryant M (2019). Activated carbon preparation from biomass feedstock: clean production and carbon dioxide adsorption. J Clean Prod.

[22] Bansal R, Goyal M (2005). Activated carbon adsorption.

[23] El Gamal M, Mousa H, El-Naas M, Zacharia R, Judd S (2018). Bio-regeneration of activated carbon: a comprehensive review. Sep Purif Technol.

[24] Vega E, Lemus J, Anfruns A, Gonzalez-Olmos R, Palomar J, Martin M (2013). Adsorption of volatile sulphur compounds onto modified activated carbons: effect of oxygen functional groups. Journal of Hazardous Materials.

[25] Molina-Sabio M, Gonçalves M, Rodríguez-Reinoso F (2011). Oxidation of activated carbon with aqueous solution of sodium dichloroisocyanurate: effect on ammonia adsorption. Microporous Mesoporous Mater.

[26] Zbigniew H, Dorota K. Chapter 8: Selective removal of heavy metal ions from waters and waste waters using ion exchange methods. In: Ayben K, editor. Ion exchange technologies. Turkey: Istanbul University; 2012. 10.5772/51040.

[27] Levchuk I, Rueda Márquez J, Sillanpää M (2018). Removal of natural organic matter (NOM) from water by ion exchange – a review. Chemosphere..

[28] Inglezakis V, Poulopoulos S (2006). Adsorption, ion exchange and catalysis.

[29] Arges C, Ramani V (2013). Two-dimensional NMR spectroscopy reveals cation-triggered backbone degradation in polysulfone-based anion exchange membranes. Proc Natl Acad Sci.

[30] Yuan Z, Li X, Hu J, Xu W, Cao J, Zhang H (2014). Degradation mechanism of sulfonated poly(ether ether ketone) (SPEEK) ion exchange membranes under vanadium flow battery medium. Phys Chem Chem Phys.

[31] Arm S, Blanchard D, Fiskum S (2005). Chemical degradation of an ion exchange resin processing salt solutions. Sep Purif Technol.

[32] Sharaf G, Hassan H (2013). Removal of copper ions from aqueous solution using silica derived from rice straw: comparison with activated charcoal. Int J Environ Sci Technol.

[33] Li P, Wang J, Li X, Zhu W, He S, Han C, Luo Y, Ma W, Liu N, Dionysiou D (2019). Facile synthesis of amino-functional large-size mesoporous silica sphere and its application for Pb^2+^ removal. J Hazard Mater.

[34] Monnier A, Schuth F, Huo Q, Kumar D, Margolese D, Maxwell R, Stucky G, Krishnamurty M, Petroff P, Firouzi A, Janicke M, Chmelka B (1993). Cooperative formation of inorganic-organic interfaces in the synthesis of silicate mesostructures. Science..

[35] Huo Q, Margolese D, Ciesla U, Feng P, Gier T, Sieger P, Leon R, Petroff P, Schüth F, Stucky G (1994). Generalized synthesis of periodic surfactant/inorganic composite materials. Nature..

[36] Bagshaw S, Prouzet E, Pinnavaia T (1995). Templating of mesoporous molecular sieves by nonionic polyethylene oxide surfactants. Science..

[37] Yang H, Coombs N, Ozin G (1997). Morphogenesis of shapes and surface patterns in mesoporous silica. Nature..

[38] Richer R, Mercier L (2001). Direct synthesis of functional mesoporous silica by neutral pH nonionic surfactant assembly: factors affecting framework structure and composition. Chem Mater.

[39] Boissière C, Larbot A, van der Lee A, Kooyman P, Prouzet E (2000). A new synthesis of mesoporous MSU-X silica controlled by a two-step pathway. Chem Mater.

[40] Tanev P, Pinnavaia T (1996). Mesoporous silica molecular sieves prepared by ionic and neutral surfactant templating: a comparison of physical properties. Chem Mater.

[41] Prouzet E, Cot F, Nabias G, Larbot A, Kooyman P, Pinnavaia T (1999). Assembly of mesoporous silica molecular sieves based on nonionic ethoxylated sorbitan esters as structure directors. Chem Mater.

[42] Mercier L, Pinnavaia T (2000). Direct synthesis of hybrid organic−inorganic nanoporous silica by a neutral amine assembly route: structure−function control by stoichiometric incorporation of organosiloxane molecules. Chem Mater.

[43] Yu C, Fan J, Tian B, Zhao D (2004). Morphology development of mesoporous materials: a colloidal phase separation mechanism. Chem Mater.

[44] Zhao D, Sun J, Li Q, Stucky G (2000). Morphological control of highly ordered mesoporous silica SBA-15. Chem Mater.

[45] Peng J, Liu J, Liu J, Yang Y, Li C, Yang Q (2014). Fabrication of core–shell structured mesoporous silica nanospheres with dually oriented mesochannels through pore engineering. J Mater Chem A.

[46] Qin Q (2015). Technology and principle for pollution adsorption by mesoporous silica-based materials.

[47] Mamonov N, Mikhailov S, Dzhungurova G, Bessudnov A, Grigor’ev D, Bedrina I, Mikhailov M (2018). Investigation of СО_2_ adsorption on amine-functionalized silicas and metal-organic polymers. Russ Chem Bull.

[48] Mercier L, Pinnavaia T (1997). Access in mesoporous materials: advantages of a uniform pore structure in the design of a heavy metal ion adsorbent for environmental remediation. Adv Mater.

[49] Zhang W, Pauly T, Pinnavaia T (1997). Tailoring the framework and textural mesopores of HMS molecular sieves through an electrically neutral (S°I°) assembly pathway. Chem Mater.

[50] Moller K, Bein T (1998). Inclusion chemistry in periodic mesoporous hosts. Chem Mater.

[51] Mercier L, Pinnavaia T (1998). Heavy metal ion adsorbents formed by the grafting of a thiol functionality to mesoporous silica molecular sieves: factors affecting Hg(II) uptake. Environ Sci Technol.

[52] Blitz I, Blitz J, Gun’ko V, Sheeran D (2007). Functionalized silicas: structural characteristics and adsorption of Cu(II) and Pb(II). Colloids Surf A Physicochem Eng Asp.

[53] Fan H, Li J, Li Z, Sun T (2012). An ion-imprinted amino-functionalized silica gel sorbent prepared by hydrothermal assisted surface imprinting technique for selective removal of cadmium (II) from aqueous solution. Appl Surf Sci.

[54] Chandra D, Das S, Bhaumik A (2010). A fluorophore grafted 2D-hexagonal mesoporous organosilica: excellent ion-exchanger for the removal of heavy metal ions from wastewater. Microporous Mesoporous Mater.

[55] Nabil M, Mahmoud K, El-Shaer A, Nayber H (2018). Preparation of crystalline silica (quartz, cristobalite, and tridymite) and amorphous silica powder (one step). J Phys Chem Solids.

[56] Zhang Q, Lee I, Ge J, Zaera F, Yin Y (2010). ChemInform abstract: surface-protected etching of mesoporous oxide shells for the stabilization of metal nanocatalysts. ChemInform..

[57] Hoffmann F, Cornelius M, Morell J, Froeba M. Silica-based mesoporous organic—inorganic hybrid materials. ChemInform. 2006;37(34). 10.1002/anie.200503075 16676373

[58] Lian M, Feng Q, Wang L, Niu L, Zhao Z, Li X, Zhang Z (2019). Highly effective immobilization of Pb and Cd in severely contaminated soils by environment-compatible, mercapto-functionalized reactive nanosilica. J Clean Prod.

[59] Li S, Jiao X, Yang H (2013). Hydrophobic core/hydrophilic shell structured mesoporous silica nanospheres: enhanced adsorption of organic compounds from water. Langmuir..

[60] Yang H, Zhang G, Hong X, Zhu Y (2004). Silylation of mesoporous silica MCM-41 with the mixture of Cl(CH_2_)_3_SiCl_3_ and CH_3_SiCl_3_: combination of adjustable grafting density and improved hydrothermal stability. Microporous Mesoporous Mater.

[61] Zhu G, Yang Q, Jiang D, Yang J, Zhang L, Li Y, Li C (2006). Synthesis of bifunctionalized mesoporous organosilica spheres for high-performance liquid chromatography. J Chromatogr A.

[62] Georgescu I, Mureşeanu M, Cârjă G, Hulea V (2013). Adsorptive removal of cadmium and copper from water by mesoporous silica functionalized with N-(Aminothioxomethyl)-2-Thiophen carboxamide. J Environ Eng.

[63] Xia K, Ferguson R, Losier M, Tchoukanova N, Brüning R, Djaoued Y (2010). Synthesis of hybrid silica materials with tunable pore structures and morphology and their application for heavy metal removal from drinking water. J Hazard Mater.

[64] Lewandowski D, Bajerlein D, Schroeder G (2014). Adsorption of hydrogen peroxide on functionalized mesoporous silica surfaces. Struct Chem.

[65] Zhou Y, He Z, Tao Y, Xiao Y, Zhou T, Jing T, Zhou Y, Mei S (2016). Preparation of a functional silica membrane coated on Fe_3_O_4_ nanoparticle for rapid and selective removal of perfluorinated compounds from surface water sample. Chem Eng J.

[66] Chatterjee S, Paital A (2017). Functionalized cubic mesoporous silica as a non-chemodosimetric fluorescence probe and adsorbent for selective detection and removal of bisulfite anions along with toxic metal ions. Adv Funct Mater.

[67] Lee J, Chen C, Cheng S, Li H (2015). Adsorption of Pb(II) and Cu(II) metal ions on functionalized large-pore mesoporous silica. Int J Environ Sci Technol.

[68] Wang Y, Zhu L, Guo B, Chen S, Wu W (2014). Mesoporous silica SBA-15 functionalized with phosphonate derivatives for uranium uptake. New J Chem.

[69] The Goldenkeys. 2020. http://www.thegoldenkeys.com.cn/. Accessed 26 May 2020.

[70] Shen S, Pan T, Liu X, Yuan L, Wang J, Zhang Y, Guo Z (2010). Adsorption of Rh(III) complexes from chloride solutions obtained by leaching chlorinated spent automotive catalysts on ion-exchange resin Diaion WA21J. J Hazard Mater.

[71] Yao S, Zhang J, Shen D, Xiao R, Gu S, Zhao M, Liang J (2016). Removal of Pb(II) from water by the activated carbon modified by nitric acid under microwave heating. J Colloid Interface Sci.

[72] Casado N, Morante-Zarcero S, Pérez-Quintanilla D, Sierra I (2018). Evaluation of mesostructured silicas with wormhole-like framework functionalized with hydrophobic groups as alternative sorbents for extraction of drug residues from food samples. Mater Lett.

[73] Wang Y, Liu Y, Zhan W, Zheng K, Lian M, Zhang C, Ruan X, Li T (2020). Long-term stabilization of Cd in agricultural soil using mercapto-functionalized nano-silica (MPTS/nano-silica): a three-year field study. Ecotoxicol Environ Saf.

[74] Fang W, Shi X, Yang D, Hu X, Williams P, Shi B, Liu Z, Luo J (2020). In situ selective measurement based on diffusive gradients in thin films technique with mercapto-functionalized mesoporous silica for high-resolution imaging of Sb(III) in soil. Anal Chem.

[75] Sharma R, Puri A, Kumar A, Monga Y, Gaba G, Adholeya A (2014). Diacetylmonoxime functionalized silica gel: an efficient and recyclable organic inorganic hybrid material for selective removal of copper from fly ash ameliorated soil samples. Sep Sci Technol.

[76] Grzesiak P, Łukaszyk J, Gabała E, Kurczewska J, Schroeder G (2016). The influence of silica functionalized with silanes on migration of heavy metals in soil. Pol J Chem Technol.

[77] Wang Y, Zhan W, Zheng K, Liu Y, Zou X, Zhang C, Ruan X (2019). Effect of surface-modified nano-silica on the mobility and fraction of Cd in contaminated agricultural soils. Soil Sediment Contam Int J.

[78] Davison W. Diffusive gradients in thin-films for environmental measurements: Cambridge University Press; 2016.

[79] Nriagu J, Becker C (2004). Volcanic emissions of mercury to the atmosphere: global and regional inventories. Sci Total Environ.

[80] Obrist D, Kirk J, Zhang L, Sunderland E, Jiskra M, Selin N (2018). A review of global environmental mercury processes in response to human and natural perturbations: changes of emissions, climate, and land use. Ambio..

[81] Risher J (2003). Elemental mercury and inorganic mercury compounds.

[82] Reis A, Davidson C, Vale C, Pereira E (2016). Overview and challenges of mercury fractionation and speciation in soils. TrAC Trends Anal Chem.

[83] Liu J, Feng X, Qiu G, Yao H, Shang L, Yan H (2011). Intercomparison and applicability of some dynamic and equilibrium approaches to determine methylated mercury species in pore water. Environ Toxicol Chem.

[84] Cattani I, Spalla S, Beone G, Del Re A, Boccelli R, Trevisan M (2008). Characterization of mercury species in soils by HPLC–ICP-MS and measurement of fraction removed by diffusive gradient in thin films. Talanta..

[85] Liu J, Feng X, Qiu G, Anderson C, Yao H (2012). Prediction of methyl mercury uptake by rice plants (Oryza sativa L.) using the diffusive gradient in thin films technique. Environ Sci Technol.

[86] Buffle J. In situ monitoring of aquatic systems: chemical analysis and speciation: John Wiley & Sons; 2001.

[87] Ridošková A, Pelfrêne A, Douay F, Pelcová P, Smolíková V, Adam V (2019). Bioavailability of mercury in contaminated soils assessed by the diffusive gradient in thin film technique in relation to uptake by miscanthus × giganteus. Environ Toxicol Chem.

[88] Huu Nguyen V, Yee S, Hong Y, Moon D, Han S (2019). Predicting mercury bioavailability in soil for earthworm eisenia fetida using the diffusive gradients in thin films technique. Environ Sci Pollut Res.

[89] Docekalová H, Diviš P (2005). Application of diffusive gradient in thin films technique (DGT) to measurement of mercury in aquatic systems. Talanta..

[90] Turull M, Fontàs C, Díez S (2019). Diffusive gradient in thin films with open and restricted gels for predicting mercury uptake by plants. Environ Chem Lett.

[91] Bratkič A, Klun K, Gao Y (2019). Mercury speciation in various aquatic systems using passive sampling technique of diffusive gradients in thin-film. Sci Total Environ.

[92] Jain C, Ali I (2000). Arsenic: occurrence, toxicity and speciation techniques. Water Res.

[93] Ferguson J, Gavis J (1972). A review of the arsenic cycle in natural waters. Water Res.

[94] Su S, Zeng X, Bai L, Wang Y, Zhang L, Li M, Wu C. Concurrent methylation and demethylation of arsenic by fungi and their differential expression in the protoplasm proteome. Environ Pollut. 2017;225:620–7. 10.1016/j.envpol.2017.03.030 28336093

[95] Bennett W, Teasdale P, Panther J, Welsh D, Zhao H, Jolley D (2012). Investigating arsenic speciation and mobilization in sediments with DGT and DET: a mesocosm evaluation of oxic-anoxic transitions. Environ Sci Technol.

[96] Wang C, Yao Y, Wang P, Hou J, Qian J, Yuan Y, Fan X (2016). In situ high-resolution evaluation of labile arsenic and mercury in sediment of a large shallow lake. Sci Total Environ.

[97] Gorny J, Lesven L, Billon G, Dumoulin D, Noiriel C, Pirovano C, Madé B (2015). Determination of total arsenic using a novel Zn-ferrite binding gel for DGT techniques: application to the redox speciation of arsenic in river sediments. Talanta..

[98] Bennett W, Teasdale P, Panther J, Welsh D, Jolley D (2011). Speciation of dissolved inorganic arsenic by diffusive gradients in thin films: selective binding of As(III) by 3-mercaptopropyl-functionalized silica gel. Anal Chem.

[99] Gorny J, Dumoulin D, Alaimo V, Lesven L, Noiriel C, Madé B, Billon G (2019). Passive sampler measurements of inorganic arsenic species in environmental waters: a comparison between 3-mercapto-silica, ferrihydrite, Metsorb®, zinc ferrite, and zirconium dioxide binding gels. Talanta..

[100] Garnier J, Garnier J, Jézéquel D, Angeletti B (2015). Using DET and DGT probes (ferrihydrite and titanium dioxide) to investigate arsenic concentrations in soil porewater of an arsenic-contaminated paddy field in Bangladesh. Sci Total Environ.

[101] Santner J, Larsen M, Kreuzeder A, Glud R (2015). Two decades of chemical imaging of solutes in sediments and soils – a review. Anal Chim Acta.

[102] Santner J, Williams P. Measurement at high spatial resolution. In W. Davison (Ed.), Diffusive gradients in thin-films for environmental measurements (Cambridge Environmental Chemistry Series, pp. 174–215). Cambridge: Cambridge University Press. 10.1017/CBO9781316442654.009

[103] Shi X, Fang W, Tang N, Williams P, Hu X, Liu Z, Yin D, Ma L, Luo J (2018). In situ selective measurement of Se(IV) in waters and soils: diffusive gradients in thin-films with bi-functionalized silica nanoparticles. Environ Sci Technol.

[104] Yin D, Fang W, Guan D, Williams P, Moreno-Jimenez E, Gao Y, Zhao F, Ma L, Zhang H, Luo J (2020). Localized intensification of arsenic release within the emergent rice rhizosphere. Environ Sci Technol.

[105] Williams P, Santner J, Larsen M, Lehto N, Oburger E, Wenzel W, Glud R, Davison W, Zhang H (2014). Localized flux maxima of arsenic, lead, and iron around root apices in flooded lowland rice. Environ Sci Technol.

[106] Cui J, Li Y, Jin Q, Li F (2020). Silica nanoparticles inhibit arsenic uptake into rice suspension cells via improving pectin synthesis and the mechanical force of the cell wall. Environmental Science: Nano.

[107] El-Naggar M, Abdelsalam N, Fouda M, Mackled M, Al-Jaddadi M, Ali H, Siddiqui M, Kandil E (2020). Soil application of nano silica on maize yield and its insecticidal activity against some stored insects after the post-harvest. Nanomaterials..

[108] Santos J, Barreto Â, Nogueira J, Daniel-da-Silva A, Trindade T, Amorim M, Maria V (2020). Effects of amorphous silica nanopowders on the avoidance behavior of five soil species—a screening study. Nanomaterials..

[109] Zhang X, Li J, Li D, Zhang H, Hu H (2020). Silicon dioxide nanoparticles have contrasting effects on the temporal dynamics of sulfonamide and β-lactam resistance genes in soils amended with antibiotics. Environ Res Lett.

[110] Liu C, Xu N, Feng G, Zhou D, Cheng X, Li Z (2017). Hydrochars and phosphate enhancing the transport of nanoparticle silica in saturated sands. Chemosphere..

[111] Wang Y, Nowack B (2018). Dynamic probabilistic material flow analysis of nano-SiO2, nano iron oxides, nano-CeO_2_, nano-Al_2_O_3_, and quantum dots in seven European regions. Environ Pollut.

[112] Carey M, Meharg C, Williams P, et al. Global sourcing of low-inorganic arsenic rice grain. Exposure Health. 2019. 10.1007/s12403-019-00330-y.

[113] Nguyen M (2020). Worldwide bans of rice straw burning could increase human arsenic exposure. Environ Sci Technol.

