# Supplementary material for: Functionalized Mesoporous Silicon Nanomaterials in Inorganic Soil Pollution Research: Opportunities for Soil Protection and Advanced Chemical Imaging
Source: Curr Pollut Rep. 2020 Jul 1;6(3):264–80. doi: 10.1007/s40726-020-00152-6 (PMC7446291; doi:10.1007/s40726-020-00152-6)
Supplement: Supplementary file 1 — (DOCX 74 kb) [file 40726_2020_152_MOESM1_ESM.docx]

**Functionalized Mesoporous Silicon Nanomaterials in Inorganic Soil Pollution Research: Opportunities for Soil Protection and Advanced Chemical Imaging**

Jia-Wei Yang**^‡^**, Wen Fang**^†^**, Paul N. Williams**^‡^,** Carlos Eduardo Eismann^§^, Amauri Antonio Menegário^§^, Lucas Pellegrini Elias^§^, Jun Luo**^†^**, Yingjian Xu^‖,^^

Supporting Information

***S1. In-situ sampling of Cr with DGT***

Chromium (Cr) is widely used for different applications such as mining, electroplating, chromium alloy production, chromium-based pigments, leather tanning, chemical synthesis and catalysts. Consequently, different species of this toxic element can be released into the environment and potentially become bioavailable (Unceta et al. 2010). Although Cr can exist in several oxidation states, only trivalent chromium – Cr(III) and hexavalent chromium – Cr(VI) are sufficiently stable in to occur in the environment. Recent studies suggest that Cr(III) is harmful to DNA in cell-culture systems, this is despite it normally considered an essential nutrient for human and animal nutrition. Cr(VI) adversely affects people health. For chronic and acute exposure, Cr(VI) is more toxic than Cr(III), and its compounds are regulated by the Dangerous Substance Directive (65/548/ECC) (Unceta et al. 2010).

In aqueous systems, the behavior of Cr(VI) and Cr(III) is mainly controlled by pH and redox potential. The high redox potential for the Cr(VI) / Cr(III) couple in acid media benefits the stabilization of Cr(III). Under basic conditions the redox potential decreases resulting in a stabilization of Cr(VI). Until pH 4 Cr(III) tends to form complexes with water, ammonia, sulphate, urea and organic acids. In a pH range of 4-6, Cr(III) tend to form hydrolysis products (Cr(OH)_n+m_). Precipitated Cr(OH)_3_(S) (a dominate form of Cr(III) in the environment) is formed at pH higher than 6. At pH above 9, Cr(OH)_3_(S) changes to the soluble Cr(OH)_4_- complex. In natural water, Cr(III) form inert precipitate at neutral pH or soluble complexes with organic ligands (Unceta et al. 2010).

In the environment, Cr(VI) can form as insoluble solid mineral associate (chromate) with other cations of Pb, Ca and Ba. Cr(VI) anions are repelled by negative charges from soils which predominate in clay minerals especially. Only manganese oxide seems to be effective in oxidizing Cr(III) to Cr(VI). On the other hand, CrO_4_^2-^ and HCrO_4_^2-^ can be easily reduced by Fe(II), phosphate, sulphite and organic material. Reijonen and Hartikainen (2016) detailed the oxidation mechanism and chemical bioavailability of Cr in agriculture soil focusing on pH as the main variable (Reijonen and Hartikainen 2016). Soils strongly influence the quality of their associated waterbodies. It remains a constant challenge to maintain the safety of potable water supplies. The World Health Organization’s stringent maximum concentration level (MCL) of total chromium in drinking water set at 50 μg L^-1^ (World Health Organization 2019), this explains why to date, chromium chemistry and speciation in particular has been most extensively studied in drinking water.

The traditional analysis methods of Cr speciation are approaches using UV-visible low-cost spectroscopy, solid-state speciation and hyphenated techniques (or on-line methods). Although the first approach is cheaper, but it is can be influenced by spectral interference, interconversion of species, losses and non-quantitative recovery. Using solid-state speciation techniques (X-ray absorption near edge structure, etc.) can avoid partially sample preparation, but is limited to relatively high detection limits. Recently, there is an agreement that hyphenated techniques are the most appropriate technique for redox speciation analysis of Cr. Flow systems coupled with atomic spectrometry have been verified to be suitable tools for automation of preconcentration/separation of Cr species. Flow systems based on the use of solid-phase extraction (the most popular approach) are fast and selective. There have also been extensively miniaturized. The various techniques are revived by Trzonkowska et al (2016). Finally, HPLC-ICP-MS is currently the most extensively used for TTEs analysis, and represents the most sensitive/versatile analytical tool for Cr speciation (Ščančar and Milačič 2014). But the powerful coupling of HPLC-ICP-MS also can’t ensure that there is no interconversion of Cr species, the main reason is the step of sampling and storage are considered in the analysis. Only in situ technique can ensure reliable sampling of Cr species. But even the most rigours preservation techniques can not completely preserve the samples, it will only ever slow down the inevitable on-going chemical and biological changes which occur after collection. Accordingly, passive samplers, (e.g. based on DGT) compared to traditional grab sampling collection methods, can be considered an effective alternative because it’s a true in-situ sampling technique.

Recently, Menegário et al. (2017) revised the use of DGT for in situ sampling of Cr species. Basically, these methods include assembling the DGT device with a binding phase known to be specific / selective for different target Cr species: a binding phase selective for anions and another binding phase selective for cations, this combination seems to satisfy the demand for research in this field. Some examples consist in combining a sodium poly (aspartic acid) solution with a DGT device as the binding phase for Cr(III) sampling, obtaining a detection limit of 3.18 µg L^-1^ and another DGT which used polyquaternary ammonium salt as binding phase for Cr(VI) sampling, reached a limit of detection of 2.92 µg L^-1^ (Guo et al. 2014). Alternatively, DGT devices assembled with a DE81 binding layer can be used for Cr(III) and Cr(VI) speciation analysis, Cr(III) is retained by Chelex-100, DE81 binding layer sampled Cr(VI) (Suárez et al. 2016). The main limitation of this approach is the low ion exchange capacity of the DE81 membrane. Thus, the use of FMSN can be an excellent way to development new DGT binding phaseS with high ion exchange capacity as these materials have high surface areas and binding capacity. In 2004, Al-Abadleh et al. reported the adsorption performance of Cr(VI) by FMSN. They found that the acid- and the ester-functionalized surfaces can bind Cr(VI) strongly. The higher the degree of surface functionalization of FMSN, the more favourable the Cr(VI) adsorption (Abadleh et al. 2004). A similar conclusion emerged from the research results of Lee et al. (2018). As the concentration of FMSN surface functional groups increased from 0.01 M to 0.25 M, the intake of Cr(VI) increased from 36.95 mg g^-1^ to 83.50 mg g^-1^ (Lee et al. 2018). These results reveal that the key factor for Cr(VI) adsorption is not the internal pore structure, but the specific functional groups. Nayab et al. (2018) used FMSN gel as an adsorbent to remove Cr(VI) from water. Results shown the FMSN gel exhibits some excellent abilities (fast adsorption kinetics, high adsorption capacity, and excellent regeneration / reusability performance, etc.) (Nayab et al. 2018). This research results shows that FMSN has the potential to selectively adsorb Cr speciation, as a DGT binding phase.

Suárez et al. (2016) reported an another analysis speciation method which used a different approach, wherein the same binding layer (zirconium gel), retains both Cr(VI) and Cr(III). NaOH was used as eluent to separate the species, which can exclusively elute Cr(VI) (Suárez et al. 2016). Although, this is approach is not as reliable as direct selective capture of species in situ, due to concerns of post-samping species conversion, with the sample stabilisation stage being of upmost importance here.


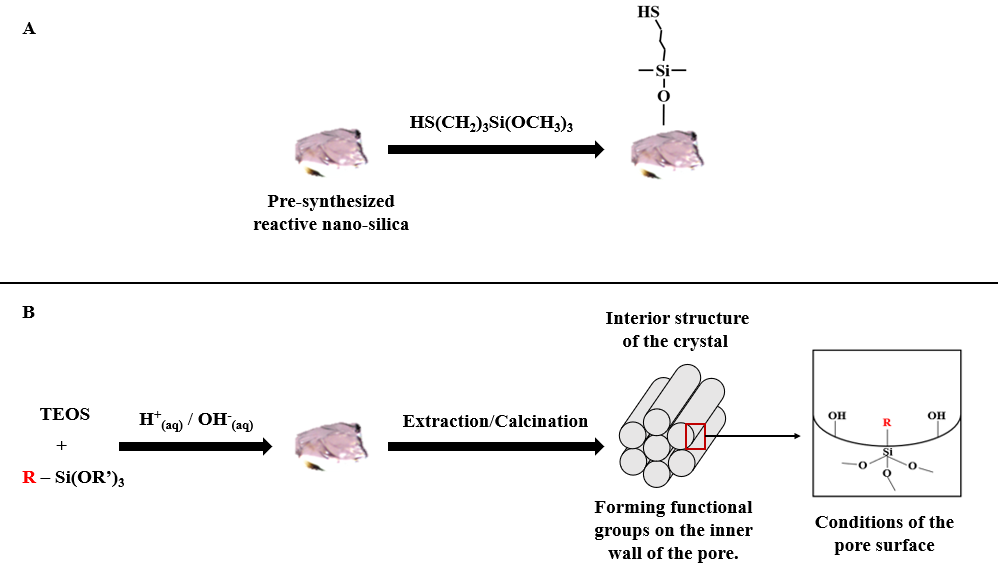


**Fig. S1.** Functionalized methods of FMSN. A). The post-synthesis surface modification of reactive nano-silica (Lian et al. 2019). B). Direct synthesis for the organic modification of mesoporous pure silica. R=organic functional group (Hoffmann et al. 2006).

References

1. Unceta N, Séby F, Malherbe J, Donard O. Chromium speciation in solid matrices and regulation: a review. Analytical and Bioanalytical Chemistry. 2010;397(3):1097-1111.
2. Reijonen I, Hartikainen H. Oxidation mechanisms and chemical bioavailability of chromium in agricultural soil – pH as the master variable. Applied Geochemistry. 2016;74:84-93.
3. World Health Organization. Chromium in drinking-water. 2019. <https://www.who.int/water_sanitation_health/publications/chromium/en/>. Accessed 29 Nov 2019.
4. Trzonkowska L, Leśniewska B, Godlewska-Żyłkiewicz B. Recent advances in on-line methods based on extraction for speciation analysis of chromium in environmental matrices. Critical Reviews in Analytical Chemistry. 2016;46(4):305-322.
5. Ščančar J, Milačič R. A critical overview of Cr speciation analysis based on high performance liquid chromatography and spectrometric techniques. Journal of Analytical Atomic Spectrometry. 2014;29(3):427-443.
6. Menegário A, Yabuki L, Luko K, Williams P, Blackburn D. Use of diffusive gradient in thin films for in situ measurements: a review on the progress in chemical fractionation, speciation and bioavailability of metals in waters. Analytica Chimica Acta. 2017;983:54-66.
7. Guo L, Chen H, Zhang Y, Bo L, Li J. Determination of chromium speciation in tap water using diffusive gradients in thin film technique. Chemistry Letters. 2014;43(6):849-850.
8. Suárez C, de Simone T, Menegário A, Rolisola A, Luko K, Gastmans D, da Conceição F, Kiang C. In situ redox speciation analysis of chromium in water by diffusive gradients in thin films using a DE81 anion exchange membrane. Talanta. 2016;154:299-303.
9. Lee J, Kim J, Choi K, Kim H, Park J, Cho S, Hong S, Lee J, Lee J, Lee S, Lee S, Choi J. Investigation of the mechanism of chromium removal in (3-aminopropyl)trimethoxysilane functionalized mesoporous silica. Scientific Reports. 2018;8(1).
10. Al-Abadleh H, Voges A, Bertin P, Geiger F. Chromium(VI) Binding to functionalized silica/water interfaces studied by nonlinear optical spectroscopy. Journal of the American Chemical Society. 2004;126(36):11126-11127.
11. Nayab S, Baig H, Ghaffar A, Tuncel E, Oluz Z, Duran H, Yameen B. Silica based inorganic–organic hybrid materials for the adsorptive removal of chromium. RSC Advances. 2018;8(42):23963-23972.
12. Lian M, Feng Q, Wang L, Niu L, Zhao Z, Li X, Zhang Z. Highly effective immobilization of Pb and Cd in severely contaminated soils by environment-compatible, mercapto-functionalized reactive nanosilica. Journal of Cleaner Production. 2019;235:583-589.
13. Hoffmann F, Cornelius M, Morell J, Froeba M. Silica-based mesoporous organic—inorganic hybrid materials. ChemInform. 2006;37(34).
